# An integrative review on the information and communication needs of parents of children with cancer regarding the use of complementary and alternative medicine

**DOI:** 10.1186/s12906-020-02882-y

**Published:** 2020-03-17

**Authors:** Trine Stub, Agnete E. Kristoffersen, Grete Overvåg, Miek C. Jong

**Affiliations:** 1 National Research Center in Complementary and Alternative Medicine (NAFKAM), Department of Community Medicine, Faculty of Health Sciences, UiT The Arctic University of Norway, Hansine Hansens veg 19, 9019 Tromsø, Norway; 2 Science and Health Library, UiT The Arctic University of Norway, Hansine Hansens veg 19, 9019 Tromsø, Norway

**Keywords:** Complementary health approaches, Complementary medicine, Paediatric oncology, Systematic review, Decision-making, Traditional medicine

## Abstract

**Background:**

Parents often choose Complementary and Alternative Medicine (CAM) as a supportive agent with the aim to reduce cancer treatment-related symptoms in their children. Therefore, it is necessary to understand parents´ information and communication needs regarding CAM. The aim of the present study was to review the research literature as to identify the information and communication needs of parents of children with cancer, and the children themselves, regarding the use of CAM.

**Methods:**

An integrative systematic review design was chosen. Searches were performed in AMED, CAMbase, CINAHL (Ebsco), EMBASE, PubMed and PsycInfo, Theme eJournals and Karger. The search was limited to studies published in English, German, Dutch, and the Scandinavian languages. Using predefined inclusion and exclusion criteria, two reviewers independently screened the title and abstracts of the relevant papers. A data extraction form and critical appraisal checklists were used to extract data for analysis, and a mixed methods synthesis was applied.

**Results:**

Out of 24 studies included, 67% were of quantitative and 33% of qualitative study design. Five main themes emerged from the analysis of 21 studies: Information on CAM, sources of CAM information, communication about CAM, informed decision-making on CAM, and Risk/benefit of CAM. The majority of the parents did not disclose the CAM use of their children because they feared negative reactions from the attending oncologist. To make informed treatment decisions for their children, parents wanted unbiased information about CAM and would act accordingly. They demand open communication about these modalities and respect for the family’s autonomy when choosing CAM for their children.

**Conclusion:**

There is an urgent need for parents of children with cancer for high quality information on CAM from reliable and scientific sources. Development of authoritative evidence-based decision tools is thus warranted to enable health care professionals and parents of children with cancer to make well informed, individual decisions concerning CAM.

## Background

The symptom burden in children with cancer is high, and is reported to increase with disease progression and cancer-related treatment [1, 2]. According to parents, symptoms such as pain, emotional distress, fatigue and loss of appetite cause the most problems for children undergoing cancer treatment [3]. Parents often choose Complementary and Alternative Medicine (CAM) as supportive agent with the aim to reduce these cancer treatment-related symptoms in their children [4]. CAM is defined as a group of diverse medical and health care systems, practices, and products that are not generally considered part of conventional medicine [5]. More specifically, if a non-mainstream practice is used together with conventional medicine, it is considered “complementary”, and if a non-mainstream practice is used in place of conventional medicine, it is considered “alternative” [5]. Reported prevalence rates of CAM use among children with cancer vary between 6 and 100% [6], depending on the definition of CAM, the sample, and country surveyed [7]. The prevalence of CAM use is on average 47.2% in high-income countries [7]. Additionally to using CAM as supportive care, parents also use CAM for treatment and cure of cancer in their children [7]. Abandonment of conventional cancer treatment in favor of CAM has been reported, and may have serious survival implications for children. A recent study in Thailand reported that the median survival duration in children who were diagnosed with acute leukemia and solely used CAM to cure cancer was 1 month, and their five-year survival rate 0% [8]. CAM modalities most commonly used in children with cancer are herbs, dietary and nutritional supplements, and spiritual treatments including faith, prayer, and healing [6, 7]. Whereas patterns of CAM use have found to be different between treatment and post-treatment in adults with cancer [9], this does not seem to appear in children with cancer. A study by Turhan et al. [10] demonstrated that there was no difference in the use of herbs or vitamins/minerals/nutrient supplements in children during chemotherapy treatment or after chemotherapy treatment.

Despite the high prevalence of CAM use in children with cancer, the majority of parents do not disclose CAM use to the attending oncologist or physician of the child [7]. Common reasons of parents not to tell about CAM are their belief that CAM is safe, fear of the physician’s reaction, that the medical staff lacks knowledge, and that the physician does not ask [7]. However, parent-physician communication on CAM use in children with cancer is of utmost importance, not alone as to prevent the risk of decreased efficacy of conventional cancer therapy because of the potential interaction with CAM modalities such as herbs and dietary supplements [11, 12]. Physicians acknowledge that it is important to know which CAM modalities their patients use, but they have little knowledge about them, and find themselves unable to inform parents about the safety and efficacy of CAM therapies [13, 14].

Other authoritative resources that parents may turn to for information are websites that advise patients on CAM use for cancer, such as the website of the National Cancer Institute [15], CAM cancer of NAFKAM (http://cam-cancer.org), and the website of the National Center for Complementary and Integrative Health (https://nccih.nih.gov). These websites however, are more directed towards cancer in adults and contain sparse to no information on the suitability of CAM modalities for use in children. Other sources that parents rely on to obtain information about CAM use are the Internet. Information from Internet is less reliable [16, 17] and can be overwhelming for parents. In addition, parents often learned about CAM from friends and family [18].

To develop future authoritative and reliable CAM resources, specifically for parents of children with cancer, it is necessary to understand which information and communications needs they have regarding CAM. Therefore, the present study was initiated with the aim to review the research literature as to identify the information and communication needs of parents of children with cancer, and the children themselves, regarding the use of CAM. To the best of our knowledge, no previous systematic review or protocol for such a planned review on information and communication needs of CAM of parents of children with cancer has been published.

## Methods

### Design and objective

There are several systematic approaches to review and synthesize the literature [19]. Given the aim of the study, an integrative review was deemed the most suitable type of review method. An integrative review is a specific review method that summarizes past empirical or theoretical literature to provide a more comprehensive understanding of a particular phenomenon or healthcare problem [20]. An integrative review allows for the inclusion of studies with different methodologies to more fully understand a particular phenomenon of concern [21]. The aim of this integrative review differs from the aim of a scoping review, which is to identify knowledge gaps, scope a body of literature, or clarify concepts, without any methodological quality assessment and integrative synthesis of the included studies [22]. This integrative review was performed in accordance with the methodology of Whittemore and Knafl [21], and the results, were applicable, were reported according to the Preferred Reporting Items for Systematic review and Meta-Analyses (PRISMA) [23]. The protocol of the integrative review was not registered in a database and involved secondary analysis of data already published in the literature. Therefore, the present study was exempt from medical ethical review.The objective and main research questions of this integrative review were guided by a Population – Concept – Context (PCC) mnemonic [24]. The population was parents, families, and/or caregivers of children with cancer, and children/adolescents with cancer themselves; the concept was the information and communication needs regarding CAM; and the context was studies of both quantitative and qualitative methodology in all types of settings. The objective of this integrative review was to identify and describe the information and communication needs of parents of children with cancer, and children/adolescents themselves, regarding CAM. The integrative review questions were: 1. Which information needs can be identified for parents of children with cancer, and/or their children? 2. Which sources do parents of children with cancer, and/or their children use to obtain CAM cancer-relevant information? 3. What needs do parents of children with cancer, and/or their children have regarding communication about CAM?

### Eligibility criteria

Eligibility criteria for inclusion and exclusion of studies were defined according to the PCC mnemonic:1. The studies included described the perspective of the parents, families, and caregivers of children with cancer, as well the perspectives of children/adolescents with cancer up to 18 years. Studies describing the perspective of healthcare professionals or CAM providers were excluded.2. The studies included described how to search and find information on CAM regarding childhood cancer and CAM information needs.

3. The studies included described the needs to communicate about CAM regarding childhood cancer with healthcare professionals, CAM providers, friends, and relatives. Studies that described information and communication needs regarding cancer in adults were excluded, as well as studies that reported solely about the patients’ and/or parents’ disclosure of CAM use (without any study data) to health care professionals.

Different quantitative and qualitative research methodologies were included such as experience reports, surveys, expert opinions, individual and group interviews, and guidelines. Studies describing all type of cancers and stages of cancer, including treatment phase, post-treatment, and palliative phase, and in all settings were included. Studies included could be published in the Danish, Dutch, English, German, Norwegian, and Swedish languages. Since authors of this integrative review had excellent understanding of these six languages, translation of included studies was not necessary. Studies that were available only in the form of abstracts or notes were excluded. Searches were not restricted to any time/date.

### Information sources and search strategy

Searches were performed by a health sciences librarian and the first author in the following databases: AMED, CAMbase, CINAHL (Ebsco), EMBASE, PubMed and PsycInfo. The German publishers Theme eJournals and Karger were also searched for studies. To identify additional studies not found by electronic searches, the reference lists of articles were checked. Depending on the database, abstracts and keywords were searched and various combinations of medical subject headings (MESH) terms and keywords were used, such as neoplasms, paediatrics child, adolescents, puberty, young adult, alternative therapies, complementary therapies, communication, information, information needs, dialogue, patient education, physician-patient relations, and dietary supplements. They were combined with “OR” and “AND” (the search-strings are attached as additional information). Keywords were adapted for the other electronic databases according to the specific subject headings or structure (see supplementary file).

### Study selection and data management

**Fig. 1:**
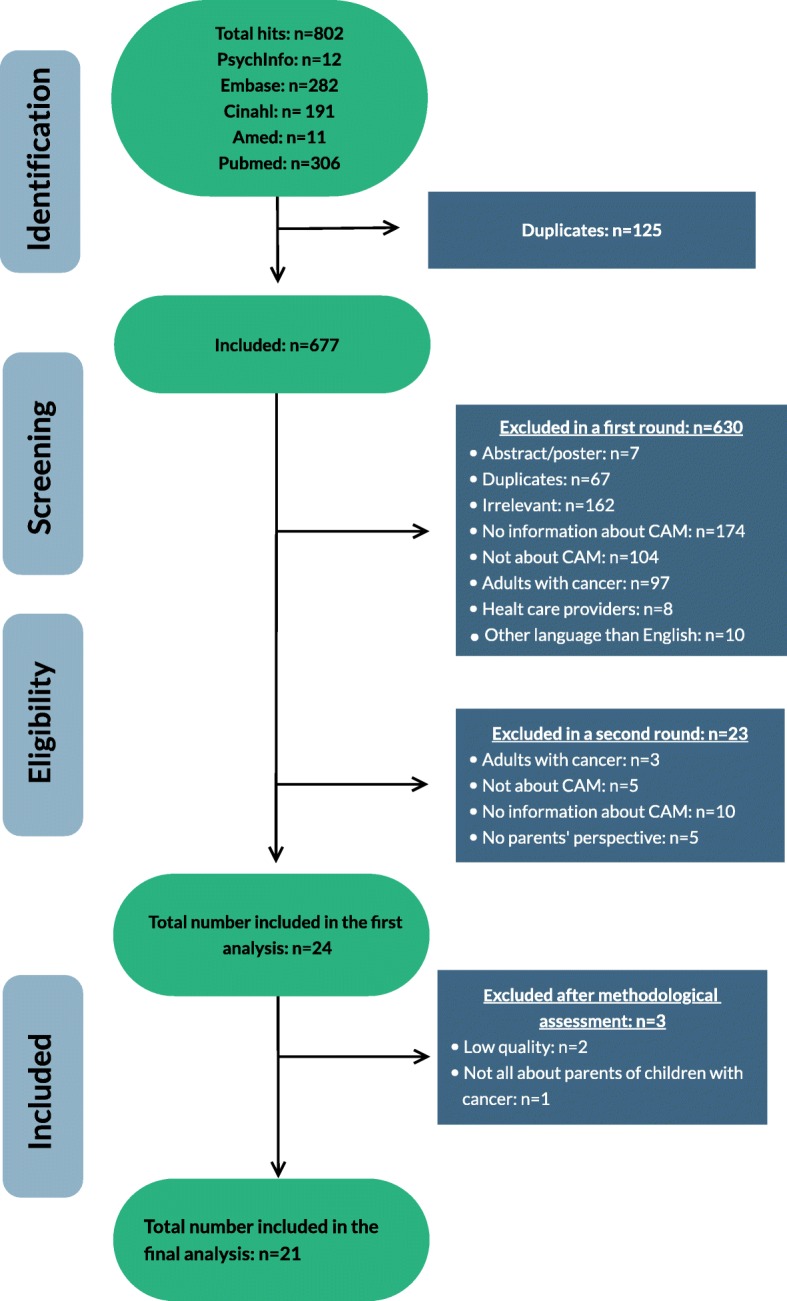
Flow chart of the inclusion process in this study

Search results were uploaded in the reference manager program Endnote to facilitate study selection, and a single data management file was produced of all references identified through the search process. Duplicates were removed and two authors screened the remaining references independently. Two authors read the included articles and extracted the data, Disagreements between the authors were discussed and solved. In two cases of disagreement between the authors, additional information from study corresponding authors was sought on the basis of which it was decided that the articles did not meet the inclusion criteria. Reasons for excluding articles were documented. Neither of the review authors was blind to the journal titles, study authors, or institutions. A flowchart of the study selection and identification according to the (PRISMA-P) guidelines [23] was generated (see Fig. 1). Two authors read the articles and extracted the data. Data was extracted from all included articles using a pre-defined data charting form. Data extracted was study characteristics such as subject of the study, methodology, study design, aim, participants, sample size, inclusion and exclusion criteria, main findings, and funding of the study.

### Appraisal of study quality

Critical appraisal tools from the Joanna Briggs Institute were used to assess the methodological quality of the included cross-sectional studies [25] and expert opinions [26]. Qualitative studies were assessed using the 10-question appraisal tool from the Critical Appraisal Skills Program (CASP) checklist [27]. The criteria used to critically appraise the quality of each study design are described in Table 2. Two review authors conducted study quality assessment, and discrepancies between the authors’ assessments were discussed and resolved. A table was generated for each quality item among all studies with the same design.

### Data synthesis

The extracted data was analyzed according to the stages as described by Whittemore and Knafl [21], using a mixed methods synthesis [50]. First, primary source data was divided by three study designs (cross-sectional studies, expert opinions, and qualitative studies), and a segregated synthesis analysis per design was performed. Data reduction was performed on predetermined thematic categories (deductive) and new appearing categories (inductive) that were relevant for the review question. Predetermined thematic categories were risk perception (risk/benefit of CAM), direct risk situations (adverse effects/ negative interactions of CAM), indirect risk situations (ethical, disease causality, and treatment philosophy), risk communication (ineffective provider-patient relationship, delay, decline conventional medicine, how to talk about the use of CAM), and information regarding CAM (lack of knowledge about CAM, where do patients gather information, unmet information needs). These categories derived from a previously performed study on perception and communication among conventional and complementary health care providers involving cancer patients’ use of CAM [51]. An additional predetermined thematic category, informed decision making, derived from a previous study on effective communication about the use of CAM in cancer care [52].

The next step was data display, converting data from individual sources into a matrix display to assemble data from multiple sources around the three study designs. Subsequently, quantitative data was translated to qualitative data to allow for a mixed methods synthesis. Constant data comparisons between the three display matrixes resulted in sub-themes and main emerging themes. The emerging themes were categorized into a table format, and discernment of main themes and sub-themes were verified with the primary source data for accuracy and transparency. Two review authors extensively discussed the outcome of each stage in the analysis process as to agree on emerging themes. Both review authors had previous experience with interviewing parents of children with cancer concerning their information and communication needs on CAM. However, both authors were aware as to not let their previous research experience affect the objectivity of data analysis and result interpretation. A record was kept during the stages of analysis as to document all thoughts, patterns, relationships, and interpretations made.

## Results

### Searches

The literature searches resulted in 802 potentially relevant studies (Fig. 1). All abstracts were screened for relevance using Endnote version X9 and the Annotated reference style. The Endnote program allowed the researcher to read the abstracts, exclude duplicates (*n* = 125), and put the studies into different categories of relevance. A total of 630 studies were excluded in a first round due to irrelevance (7 studies were abstracts, 67 studies were duplicates, 162 studies were irrelevant, 174 had no information about CAM, 104 studies were not about CAM, 97 studies included adults with cancer, 8 studies included health care providers, 10 studies were in languages other than English, German, Dutch, Swedish, Danish, and Norwegian, and 1 study used the same data set as another included study). In a second screening round, 23 studies were excluded (3 studies included adults with cancer, 5 studies were not about CAM, 10 studies had no information about CAM, 5 studies had not included the parents’ perspective). A total of 24 studies (Fig. 1) were included in this review [10, 18, 28–49], *n* = 16 surveys [10, 18, 28–41], *n* = 5 expert opinions [42–46], and *n* = 3 qualitative studies [47–49]. The characteristics of included studies are tabulated in Table 1.

**Table 1:** Characteristics of the included studies

Study ID	Subject/Population	Method	Design	Setting	Aim (s)	Participant(s)	Sample size	Inclusion/exclusion criteria	Results	Funding
**Agapito J, 2000** [42]	Ethical issues of declining conventional cancer treatment	Text and opinion paper, based on a case	NA	Hospital	NA	13 years boy with osteosarcoma with lung metastasis	*n* = 1	NA	The obligation of conventional health care providers to treat people holistically, and the need for them to delve more deeply into the philosophical underpinnings of parents viewpoints	NR
**AL-Qudimat, MR, 2011** [28]	Use of CAM among children with cancer in Jordan	Questionnaire and interviews with parents	Descriptive cross-sectional design	A paediatric oncology department in Jordan	To examine the use of CAM among children with cancer, including prevalence, CAM modalities used, reason for use and parent-perceived benefit of CAM	Parents of children with cancer	*n* = 84	0–18 years of age; diagnosed with cancer at least 2 months prior to the study with parents that agreed to participate in the study	Nearly half of the participants (45,5%) that used CAM perceived benefits from CAM. Parents use CAM to support their child’s medical treatment and to use all possible methods to cure their child. Most have not discussed this use with the medical staff	No funding
**The American Academy of Paediatrics, 2001** [43]	Children with chronic illness and disabilities including cancer	Recommendations for clinicians/paediatricians	Guidelines	USA	To provide information and guidance for paediatricians when consulting families about CAM	NA	NA	NA	It is important to maintain a scientific perspective to provide balanced advice about therapeutic options, to guard against bias, and to establish and maintain a trusting relationship with families	NR
**Ball SD, 2005**^**a**^ [29]	The nature and prevalence of dietary supplement use among chronically ill children	Self-reported questionnaire	Survey	Sub speciality medical clinics in South Lake City, Utah	Investigate the nature and prevalence of dietary supplement use as an adjunct to conventional medical treatment in chronically ill children	Parents of chronically ill children	*n* = 505, of which *n* = 100 with cancer	Parents with chronically ill children that were accompanying their child on the clinical visit in the period February to august, 2001.	Dietary supplements use is prevalent among chronically ill children especially among those with poor diagnosis or for whom are limited conventional medical treatment. The physicians were not informed about their patients use of dietary supplements	NR
**Ben Arush, 2006** [30]	Prevalence, types and characteristics of CAM use	Face to face questionnaire	Cross-sectional study	Paediatric haematology oncology department at Meyer Children’s hospital in Rambam medical centre, Israel	To evaluate the prevalence, types and characteristics of CAM use by ethnic demographic socio-economic and disease factors as well as involvement of the treating physicians	Parents of children with cancer, or adolescents with cancer	*n* = 74 parents and *n* = 26 adolescents	All patients/parents who visited the clinic during the last half of 2003. Patients with benign teratoma and cranio-pharyngioma were excluded	Most participants were interested in receiving information on CAM and the availability of CAM in the hospital. Most used CAM without informing their physician.	NR
**Bold J, 2001**^**a**^ [47]	Children with cancer	Semi-structured telephone interviews	Qualitative study	Region of Saskatchewan, Canada	To estimate the preference of unconventional therapy use, including identification of the commonly used therapies and to describe expectations and experiences of families seeking unconventional therapies	Parents of all children with cancer	*n* = 44	Parents of all children who were aged 14 years or younger when diagnosed with cancer during 1994 and 1995 in Saskatchewan and still living at the time of the study	36% of the participants reported using CAM, 21% considered it. Parents identified a need for better quality information about CAM	National Cancer Institute of Canada; The Canadian Cancer Society; L. Schulman Trust Fund
**Clayton MF, 2006** [44]	Brain tumour in a 10 year old boy	Text and opinion paper based on a case	NA	NA	Describe the importance of good patient-care provider communication, based on nursing a 10 year old boy who died at home (palliative phase)	NA	NA	NA	Communication by using every opportunity to learn and lessen following up on concerns, and delivering information in a way that preserves their (parents) hope and respect their decisions	NR
**Fernandez C, 1998** [31]	Paediatric patients diagnosed with cancer	Questionnaire	Retrospective cohort study	Tertiary care paediatric oncology Centre in British Columbia	To examine the use of CAM therapies in paediatric oncology patients	Parents of children with cancer	*n* = 366	Parents of children in whom a diagnosis of cancer was made between June 30, 1989 and July 1, 1995. Patients who died within 1 week of diagnosis, patients with Langerhans’ cell histiocytosis, bening teratomas and craniopharyngioma were excluded	42% used CAM. Herbal teas, plant extracts and vitamins was the most used. Factors that influenced CAM use were prior use, positive attitude towards CAM, information from families and friends or from CAM practitioners	NR
**Fletcher PC, 2004** [48]	Children diagnosed with cancer	Phone interviews	Qualitative study	Ontario, Canada	To interview parents about the experience they faced when coping with a child who has been diagnosed with cancer, with focus on the use of CAM therapies	Parents of children with cancer	*n* = 29	Parents whose children have been diagnosed with cancer within the previous 5-year period. Parents who lost their child, or of which the child was in the palliative stage were excluded	Three themes 1: Parent’s opposition to CAM utilisation. 2. Parents support of CAM use with their children with cancer 3. Physicians views of CAM as perceived by parents	NR
**Gagnon EM, 2003** [32]	Paediatric oncology patients	Questionnaire	Cross-sectional survey	Paediatric oncology clinic in USA	To investigate how parents preferred level of control in treatment decision making is related to their personal health care involvement and their decision to use CAM for their child	Parents or caretakers of paediatric patients	*n* = 118	English speaking parents of paediatric patients, accompanying a child for an appointment, the child had been diagnosed at least 1 month prior, and was either receiving treatment or had completed treatment less than 2 years prior. Parents accompanying children for consultations or emerging medical visits were excluded	Most parents preferred active or collaborative decision-making. Preference for control in decision making was not associated with CAM use	The Agency for Healthcare Research and Quality, The Leonard P. Zakin Center for Integrated Therapies at the Dana-Farber Cancer Institute
**Gilmour J, 2011** [45]	Adverse effects of chemotherapy in a 6 years old boy diagnosed with medulloblastoma	Expert opinion based on a case study	NA	NA	To explain clinicians obligations when obtaining inform consent to treatment and discuss the clinician’s responsibility to tell parents or patients about a potential beneficial CAM therapy	A 6 year old boy	NA	NA	Physicians have an ethical duty to beneficence, to do good and not harm. They must be aware of current research on pain and symptom management and other aspects of care. This may include CAM therapies when there is reliable evidence of therapeutic benefit	SikKids foundation, Alberta heritage Foundation for Medical Research, The Canadian Institutes of Health research.
**Gozum S, 2007** [33]	Childhood cancer	Semi-structured questionnaire (face to face interviews based on the questionnaire)	Cross-sectional study	Paediatric oncology unit in eastern Turkey	To gain insight into the prevalence and factors related to the use of CAM	Parents of children with cancer	*n* = 88	Parents of children with cancer who were treated at the centre and had children between 0 and 18 years of age, at least 2 months post diagnosis and agreed verbally to participate	48, 9% used CAM. Most common was herbal products/tea/meal	NR
**Krogstad T, 2007** [49]	Childhood cancer	Focus group interviews	Qualitative study	Hospital	Investigate the perceptions and experiences about dietary supplements and natural products among parents of children with cancer. From whom they prefer to receive information from and how they perceive advice from family and friends	Parents of children with cancer	*n* = 21	NR	Parents were very restrictive in giving natural products to their children. However, many parents felt considerable pressure from media, commercial advertising and the health food market to give such products to the child. Recommendations from friends and family were difficult to handle because failure to follow up advice felt like a burden. The parents did not receive information from their physician about these products	NR
**Ladas EJ, 2014** [34]	Cancer diagnosis in children and adolescents	Questionnaire	Cross-sectional study	Paediatric oncology centre in Guatemala City	To describe the prevalence, pattern of use and descriptive associations of TCAM^b^ use among children diagnosed with cancer	Parents of children and adolescents	n = 100	Parents of children and adolescents who were undergoing treatment for cancer, receiving palliative care, or have completed treatment	90% of parents reported use of TCAM. 67% used TCAM for supportive care and 34% for curative purposes. Most use was oral supplements	NR
**Laengler A, 2008** [35]	Cancer diagnosis under the age of 15	Questionnaire	Retrospective survey	All German hospitals that reported to the German Childhood Cancer Registry	To compare the group of homeopathy users with users of other CAM therapies with regard to pattern of CAM, and the attending circumstances of previous experiences of CAM	Parents of children with cancer	*n* = 367	All parents in Germany with a child under the age of 15 diagnosed in 2001 with cancer that was recorded by the German childhood cancer registry. Exclusion: Death of a child within the first 8 weeks after the diagnosis and development of a second cancer	35% of the respondents had used CAM. Sources of information about CAM were in most cases no doctors. 71% of the users had communicated the use of CAM with the doctor	The Deutsche Kinderkrebsstiftung Bonn
**Magi T, 2015** [18]	Children and adolescents with cancer	Questionnaire	Retrospective survey	University Children Hospital Bern, Switzerland	To collect information on CAM use by childhood cancer patients	Families of children with cancer	*n* = 123	All patients between 0 and 18 years who were diagnosed between January 1, 2002 and December 31, 2011, and registered in the Swiss childhood cancer Registry (SCCR). Exclusion: Death within 2 months of diagnosis and parents refusal to participate in a survey	53% had used CAM, 25% of patients received information about CAM from medical staff. Most frequent reason for not using CAM was lack of information	Swiss pediatric oncology group; Schweizeriche konferenz der kantonalen gesundheitdirektorinnen und-direktoren; Swiss Cancer Research; Kinderkrebshilfe Schweiz; Ernst-Göhner Stiftung; Stiftung Domarena, CSL Behring
**Molassiotis A, 2004** [36]	Children with cancer between 5 and 18 years of age	Questionnaire	Cross-sectional study	A large hospital in the UK	To determine the preference of CAM use among children with cancer and motives of parents for doing so	Parents of children with cancer	*n* = 49	Parents of children who have been diagnosed with cancer between November 1999 and November 2001	32,7% reported CAM use. Most used were multivitamins, aromatherapy and massage. Parents identified a need for more information	NR
**Ndao DH, 2013**	Childhood cancer survivors	Interviewer (in person or phone) based questionnaire	Survey	Herbert Irving Child and Adolescent, Oncology centre, Columbia University medical centre, NY	To investigate the prevalence of CAM use, types and reason for use, and the determinants of use among survivors of childhood cancer	Children, adolescents and young adults	*n* = 197	Children, adolescents and young adults visiting the Herbert Irving Oncology Centre, all participants were at least 3 months from completion of treatment for cancer	58% reported using CAM, 72% of which used biologically based therapies (supplements, nutrition, vitamins, minerals, herbs). 51% of all CAM therapies were disclosed to the physician	Tamarind Foundation
**Olbara G, 2018** [38]	Health care providers perspectives on TCAM in Kenya	Questionnaire	Cross-sectional study	MTRH, the largest public tertiary hospital in Western Kenya	To explore health care providers perspectives onTCAM involved in the care of children with cancer, personal experience with TCAM, health beliefs, components of TCAM, recommending or discouraging TCAM, communication between health care providers, parents and TCAM practitioners and knowledge about TCAM	Health care providers	*N* = 155	All health care providers identified from the hospital employed registry	Health care providers felt that communication with parents about TCAM should be emphasised and were enthusiastic to improve their knowledge about it.	NR
**Rajanandh MG, 2018**^**a**^ [39]	Paediatric cancer	Questionnaire	Survey	Tertiary care South Indian Hospital	Investigate the prevalence of CAM use among paediatric cancer patients in a tertiary care hospital	Male and female patients less than 18 year of age, or their parents	*n* = 277	Patients less than 18 year of age clinical diagnosed with any type of cancer for more than three months. Patients with any comorbidities were excluded	7, 6% used CAM, most common Aryurveda. None of the parents disclosed the CAM use to the oncologists	NR
**Singendonk M, 2013** [40]	Paediatric cancer	Questionnaire	Prospective multicentre study	Six academic hospitals in the Netherlands	Investigate the prevalence of CAM use, possible determinants of use, parental attitude towards communication and research on CAM therapies	Parents of children with cancer	*n* = 304	Parents of children with cancer age 0–21 with a diagnosis of malignancy in the past 5 years, attending the oncology outpatient clinics from June 2011 to January 2012. Parents with insufficient knowledge of the Dutch language were excluded	42,4% had used CAM the past 12 months. 19,1% had used more than one type of CAM, 26.5% had used over the counter products. 75% found CAM modalities effective	No funding
**Susilawati D, 2016** [41]	Cancer diagnosis in children	Questionnaire	Cross-sectional study	Paediatric department of a hospital in Indonesia	Explore and compare perspectives (parents and health care professionals) on CAM in children with cancer	Caretakers of children with cancer	*n* = 176	All parents of children with cancer who were hospitalized or visited the clinic from September 2013 to October 2014	54% think that CAM may be helpful in cancer treatment of children. Most recommended CAM was prayer (93%). Health care providers and parents had different perspective on CAM use in children with cancer	Noord-Zuid Programma, The Stichting Medicines for ALL and the doctor 2 Doctor Program
**Tautz C, 2005** [46]	Children with cancer	Expert opinion	NA	Child and adolescence medicine department of the general hospital Herdecke, Germany	To provide an overview of the CAM methods most commonly used in the treatment of children with cancer	Parents and doctors	NA	NA	The theoretic bases for anthroposophical medicine, homeopathy, phytotherapy, Boswellia preparations, vitamins, enzymes, diary supplements and psycho-oncology are described	NR
**Turhan AB, 2016** [10]	Childhood cancer	Questionnaire	Cross-sectional study	Outpatient paediatric oncology clinic	To explore the frequency of CAM use, factors affecting the use, and the individual CAM treatments used, the perception of families regarding the efficacy and safety of CAM and their sources of information	Parent of children with cancer	*n* = 74	Children undergoing chemotherapy or who have completed courses of chemotherapy, between 0 and 18 years, in the last three months post diagnosis, and parents agreed verbally to participate. Children in palliative care, had died, or had second cancers were excluded	67.5% used these products, main source of information was internet, used CAM without information healthcare professionals, incorporate it into conventional care	NR

*NA* Not applicableM, *NR* Not Reported, ^**a**^Excluded from further analysis due to either poor methodological quality or other bias, ^b^*TCAM*, Traditional CAM

### Assessment of methodological quality/critical appraisal

Five (*n* = 5) cross-sectional studies were assessed as low risk of bias as they had addressed nine out of nine items in its design, conduct, and analysis [31, 34, 38, 40, 41] (see Table 2). Three cross-sectional studies (*n* = 3) [10, 18, 28] addressed eight items, six studies (*n* = 6) [30, 32, 33, 35–37] addressed seven items, one study (*n* = 1) [29] addressed six items, and one study (*n* = 1) [39] addressed only four out of nine items. Three (*n* = 3) expert opinion papers [42, 45, 46] were assessed as low risk of bias as they had addressed all six items in its design, conduct, and analysis. Two expert opinion papers (*n* = 2) [43, 44] had addressed five out of six items. One qualitative study (*n* = 1) [48] was assessed with low risk of bias as it had addressed eight out of ten items in its design, conduct, and analysis. One study (*n* = 1) had addressed six items [49] and one study (*n* = 1) [47] addressed only four out of ten items. Of *n* = 24 studies, *n* = 12 were rated as high methodological quality (low risk of bias), *n* = 10 were rated as medium methodological quality, and *n* = 2 were rated as low methodological quality.

**Table 2:** Assessment of the methodological quality of each included study

	1. Sample representative for the target population?	2. Sample included in the study in a valid and reliable way?	3. Adequate explanation whether the respondents differed from non-responders?	4. Is there an acceptable response rate (70% or above)?	5. Are measurements appropriate?	6. Is data collection standardized?	7. Is data analysis standardized?	8. Are the results relevant for clinical practice?	9. Are the results in line with other available studies?	
**Surveys**^**a**^										
*AL-Qudimat, MR, 2011* [28]	**+**	**+**	**–**	**+**	**+**	**+**	**+**	**+**	**+**	
*Ball SD, 2005* [29]	**+**	**+**	**?**	**?**	**–**	**+**	**+**	**+**	**+**	
*Ben Arush, 2006* [30]	**+**	**+**	**?**	**?**	**+**	**+**	**+**	**+**	**+**	
*Fernandez C, 1998* [31]	**+**	**+**	**+**	**+**	**+**	**+**	**+**	**+**	**+**	
*Gagnon EM, 2003* [32]	**+**	**+**	**?**	**?**	**+**	**+**	**+**	**+**	**+**	
*Gozum S, 2007* [33]	**+**	**+**	**?**	**–**	**+**	**+**	**+**	**+**	**+**	
*Ladas EJ, 2014* [34]	**+**	**+**	**+**	**+**	**+**	**+**	**+**	**+**	**+**	
*Laengler A, 2008* [35]	**+**	**+**	**?**	**–**	**+**	**+**	**+**	**+**	**+**	
*Magi T, 2015* [18]	**+**	**+**	**+**	**–**	**+**	**+**	**+**	**+**	**+**	
*Molassiotis A, 2004* [36]	**+**	**+**	**–**	**–**	**+**	**+**	**+**	**+**	**+**	
*Ndao DH, 2013* [37]	**+**	**+**	**–**	**?**	**+**	**+**	**+**	**+**	**+**	
*Olbara G, 2018* [38]	**+**	**+**	**+**	**+**	**+**	**+**	**+**	**+**	**+**	
*Rajanandh MG, 2018* [39]	**+**	**?**	**–**	**?**	**–**	**+**	**+**	**+**	**–**	
*Singendonk M, 2013* [40]	**+**	**+**	**+**	**+**	**+**	**+**	**+**	**+**	**+**	
*Susilawati D, 2016* [41]	**+**	**+**	**+**	**+**	**+**	**+**	**+**	**+**	**+**	
*Turhan AB, 2016* [10]	**+**	**+**	**?**	**+**	**+**	**+**	**+**	**+**	**+**	
	**1. Is the source of the opinion clearly identified?**	**2. Does the source of the opinion have standing in the field of expertise?**	**3. Are the interests of the relevant population the central focus of the opinion?**	**4. Is the stated position the result of an analytical process?**	**5. Is there reference to the extant literature?**	**6. Is any incongruence with the literature/sources logically defended?**				
**Expert opinions**^**b**^										
*Agapito J, 2000* [42]	**+**	**+**	**+**	**+**	**+**	**+**				
*The American Academy of Paediatrics, 2001* [43]	**+**	**+**	**+**	**+**	**+**	**+**				
*Clayton MF, 2006* [44]	**+**	**+**	**+**	**+**	**+**	**?**				
*Gilmour J, 2011* [45]	**+**	**+**	**+**	**+**	**+**	**+**				
*Tautz C, 2005* [46]	**+**	**+**	**+**	**+**	**+**	**+**				
	**1. Was there a clear statement of the aims of the research?**	**2. Is a qualitative methodology appropriate?**	**3. Was the research design appropriate to address the aims of the research?**	**4. Was the recruitment strategy appropriate to the aims of the study?**	**5. Was the data collection in a way that addressed the research issue?**	**6. Is relationship between researcher and participants adequately considered?**	**7. Have ethical issues been taken into consideration?**	**8. Was the data analysis sufficiently rigorous?**	**9. Is there a clear statement of findings?**	**10. Is the research valuable?**
**Qualitative studies**^**c**^										
*Bold J, 2001* [47]	**+**	**?**	**+**	**+**	**?**	**–**	**?**	**–**	**–**	**+**
*Fletcher PC, 2004* [48]	**+**	**+**	**+**	**+**	**+**	**+**	**–**	**?**	**+**	**+**
*Krogstad T, 2007* [49]	**+**	**+**	**+**	**+**	**+**	**–**	**–**	**–**	**+**	**–**

(+) = Yes

(−) = No

(?) = Uncertain/Unable to assess

Assessed according to the following checklists: ^a^The Joanna Briggs Institute (2017): Critical Appraisal tools for use in JBI Systematic Reviews. Checklist for Analytical Cross Sectional Studies. ^b^The Joanna Briggs Institute (2017): Critical Appraisal tools for use in JBI Systematic Reviews. Checklist for Text and Opinion, and ^c^Critical Appraisal Skills Programme (CASP) checklist for qualitative research (accessed 2019)

### Exclusion of studies for further analysis

Three (*n* = 3) out of the 24 included studies were excluded from further analysis [29, 39, 47]. The article of Ball et al. [29] was about the use of dietary supplements by children with a chronic illness. However, only 20% of the total respondents were parents of children with cancer. Therefore, the results cannot be generalized to communication and information needs of parents of children with cancer specifically. The studies of Rajanandh and Bold et al. [39, 47] were excluded from further analysis due to low methodological quality and thereby high risk of bias (see Table 2). Therefore, twenty-one studies were used for further analysis in this review (see Fig. 1).

### Main themes

The data was organized in five main themes. Three of the five emerging themes directly related to the three integrative review questions: *Information on CAM* (review question 1), s*ources of CAM information* (review question 2), *and communication about CAM* with four sub-themes (respect, decline of conventional medicine, hope and control, disclosure of CAM use) (review question 3). Another emerging theme that related to review question 1 was *informed decision-making on CAM.* The fifth main theme observed was *risk/benefit of CAM*. “CAM use” refers to the use of CAM among children with cancer.

#### INFORMATION on CAM

Having a child with cancer is time consuming and many parents do not have the time to find reliable information about CAM. This review found that there is an apparent need among parents of children with cancer for information on CAM [40]. Fernandez [31] and Molassiotis et al. [36] reported that the most common reason for not using CAM was lack of information. In a survey, Ben-Arush et al. [30] found that the majority of the parents were interested in obtaining more information and guidance about CAM.

Many parents wanted information on CAM from authoritative sources such as the oncologist or hospital where they are treated. Singendonk et al. [40] reported that parents wanted information on CAM to be provided by the treatment oncologist and offered in the hospital. If CAM treatment had been offered in the hospital, they would have considered it for their child [30, 36]. This is in accordance with a Norwegian study where the parents would have liked to receive information from the hospital staff or the physician [49]. They would also have liked to receive information from the pharmacist, because they believed this was a more impartial source. This is in line with Fletcher et al. [48] who found in a qualitative interview study that parents wanted information about CAM integrated in the services they already received in the hospital, as lack of time hindered them to examine CAM modalities themselves.

Susilawati et al. [41] acknowledged that parents know little about CAM. This study found that the majority of the parents reported that their knowledge about the safety and efficacy of CAM was inadequate, and they wanted to learn or read more about it.

#### Sources of CAM information

Many parents want CAM information that is easily accessible, preferably from authoritative sources. However, according to nine studies (*n* = 9), the sources of information about CAM were mostly (i) *family and friends*, (ii) *health care providers (CAM and conventional),* and (iii) *the media* [10, 31, 33, 35–37, 45, 49]. (i) *Family and friends*: Fernandez et al. [31] reported that parents of children with cancer received information on CAM from *families and friends.* This is in accordance with Gozum et al. [33] who reported that most parents in Turkey learn about CAM from *friends and relatives* or *other families with children who have cancer*. According to Gilmour [45], the reason for using family and friends as the main source of information was that they did not receive CAM information from the oncologists. Children and young adults attending an oncology clinic in New York received information from their *parents* [37]. Krogstad et al. [49] found that parents received information about supplements/herbs from *friends, families, commercials,* and *natural health food stores*. The parents were, however, sceptical towards commercials regarding these products. They regarded information from families and friends trustworthy, but their advice felt like a burden because of failure to follow up. This was also the case for families in Turkey where the most frequent source of information about CAM reported by families was *the Internet* and *relatives* [10]. *Family* and *friends* were also the main source of information for the majority of the parents in Germany [35] and the UK [36].

(ii) *Health care providers (CAM and conventional)*: In addition to the parents, children and young adults attending an oncology clinic in New York also received information from *integrative providers* [37]. In Germany [35] the source of information about CAM was in most cases *health care providers* other than doctors. Fernandez et al. [31] who investigated the use of CAM reported that the factors that influenced the CAM use were prior CAM use and positive attitude towards CAM, in addition to information from CAM providers. Molassiotis et al. [36] reported that *health care providers* were the second most commonly used source of information in the UK. However, in Switzerland Magi et al. [18] found that patients mainly received information on CAM from *the medical staff* at the University hospital in Bern. In addition, Ladas et al. [34] reported that a source of information about TCAM in Guatemala City was *doctors* and *health care professionals*.

(iii) *The media:* Even though many parents wanted information on CAM from reliable sources, they often used less reliable sources such as the media. Fernandez and Thuran et al. [10, 31] reported that one third of the participants in their study received information from this source. This was in line with Laengler and Ndao et al. [35, 37] who found that *the media* was one out of three sources where parents gathered information on CAM. Molassiotis et al. [36] reported that the parents identified a need for more information and that the media was the most commonly used source of information.

#### Communication about CAM

For better quality of care, communication about CAM use is needed between parents of children with cancer and their conventional providers. According to the American Academy of Paediatrics, CAM may improve the quality of life and address specific concerns of the child and family regarding cancer [43]. Therefore, a conversation about CAM may avert feelings of frustration and powerlessness that impel families to these modalities.

##### Respect

Health care providers provide ethical care by respecting parents’ choice on using CAM for their child. They have an ethical obligation to do good, and respect the parents’ needs to try everything for their child. A team of nurses managed to maintain open communication with a mother of a severely ill son [44]. The mother informed them about the supplements the boy used, and the nurses were able to anticipate adverse interactions with other medications. She responded to the nurses’ willingness to listen by keeping them informed about her son’s CAM treatment.

##### Decline of conventional medicine.

Autonomy and the role of the family to decide treatment for their child are vital ethical dilemmas when it comes to declining conventional medicine. These issues must be handled with care by health care providers and the government, as different philosophic worldviews between parents and health care providers may lead to a decline of conventional medicine [42]. Agapito described a case where the social services department went to court to protect a 13-year-old boy from his father’s influence. The family was religious and believed that faith alone could save him. The boy rejected conventional medical care, and the social services went to court to overrule this decision. During the legal process, the boy was diagnosed with lung metastasis and the case was dropped. Consensus regarding the choice of treatment can usually be reached, but it requires time and willingness to communicate. However, the decision to go to court created a communication barrier with the family and thereby the valuable consensus and share purpose usually found between health care providers and their patients and families broke down [42]. Agapito pointed out the ethical obligation of conventional health care providers to treat people holistically and do good [44]. They need to delve more deeply into the philosophical underpinnings of the parents’ viewpoints.

##### Hope and control

The importance of keeping hope alive when the child is serious ill is important. Loss of hope creates despondency or desperation, and parents need to maintain some sense of control over life, and hope against possible death of their child. Health care providers provide ethical care by respecting parents’ choice on using CAM for their child [44]. The importance of keeping hope alive when the child was serious ill was addressed by three studies in this review [28, 42, 44]. To maintain hope, parents often perceived CAM safer and more efficient than research demonstrated, as to have rationale for trying all possible treatment methods for their child [28, 44]. Information about CAM gave a sense of control of the child’s treatment for the parents. It also provided additional ways of helping their child to get through his/her cancer treatment. Moreover, it gave parents the feeling that they were doing everything possible to support their child’s recovery [36]. Gagnon and Recklitis [32] investigated how the parents’ preferred level of control in treatment decision making was related to their personal health care involvement and their decision to use CAM for their children. They found that most parents using CAM preferred active or collaborative versus passive decision-making. Preference for control in decision-making was not associated with CAM use.

##### Disclosure of CAM use

This review found that patients want an open and non-judgmental communication about CAM with their health care provider. Qudimat et al. [28] reported that a minority of CAM users discussed the use of CAM with their health care providers. The reason for non-disclosure was that the parents thought it was not important. Others thought they would receive negative reactions from the physician, and a few stated it was because the doctor did not ask. Gozum et al. [33] reported that one third of the CAM users discussed the topic with doctors and nurses and that the majority did not. Ben-Arush [30] reported that more than half of the CAM users had never consulted or discussed CAM use with any of their physicians, oncologists, or nurses. Half of the participants, who asked the physician about CAM, reported that the physicians encouraged CAM use or proposed a consultation with a physician well trained in CAM. A minority decided not to use CAM even though they asked for advice. The health care providers raised the issue of CAM in only four cases. Singendonk et al. [38] found that in the majority of the cases the parents initiated the discussion about CAM use. The reactions from the health care providers were impartial or positive, and none had experienced negative reactions. Magi et al. [18] reported that more than half of the parents reported to have told the attending oncologist about CAM. Laengler et al. [35] found that most parents spoke to a doctor about the use of CAM. This is in accordance with Fernandez et al. [31] who reported that only a few of the parents were uncomfortable discussing CAM with their oncologist. The majority of the parents felt that their oncologist had no opinion about CAM or had not made their opinion known. Susilawati et al. [41] found that half of the parents would not raise the topic of CAM if they perceived the doctor as sceptical. Despite caution and scepticism, doctors should facilitate an atmosphere of openness in the consultation so that the patients feel comfortable discussing CAM. The parents perceived that a more open doctor-parent communication about CAM might enhance the doctor’s knowledge of which CAM modalities patients were using.

According to a study by Fletcher et al. [48], parents emphasized that the physicians should be receptive to CAM to obtain support for their choice. The health care providers, on the other hand, were cautious about CAM use during conventional cancer treatment and wanted to be informed prior to the administration of CAM. Then they could determine whether the modalities in question were appropriate. According to the parents, the physicians advised them not to initiate any treatment without informing their health care team. Krogstad et al. [49] found that if parents asked the physicians about possible use of multivitamins for the child, the physicians answered that they should decide themselves. Ndao et al. [37] reported that the disclosure rate of CAM use among children and adolescents to the physician increased from 1998 to 2008. According to the American Academy of Paediatrics [43], clinicians should listen carefully and acknowledge the families’ concerns, priorities, and fears, including social and cultural factors. They must continue to care for the family even though they choose to use CAM. This is in line with Gilmore [45] who pointed out the physician’s obligation to do good, and be updated on beneficial CAM treatment options.

#### Informed decision-making on CAM 

It is important that parents get easily accessible and reliable information about CAM to make informed decisions about these modalities. Agapito and Fernandez pointed out the importance of autonomy of the family when making treatment decisions for children [31, 42]. Adequate understandable information may empower parents and give them freedom to act on that information [31]. Clyton [44] disclosed a story about a mother who preferred to make decisions about CAM independently after receiving information from the health care team. Molassiotis et al. [36] argued that doctors and nursing staff should offer and discuss CAM with parents so that they are able to make informed decisions and that CAM literature should be available at the ward. This is in line with Tautz et al. [46] who reported that to support parents in decision making, the doctor needs to build a trustworthy and resilient relationship with the parents. They must do that without judgement. To make informed consent, parents should be informed about the placebo effect and the need for conducting controlled studies. Tautz et al. [46] proposed to make a decision aid with scientific content because there is lots of information out there that is hard to assess for the parents.

#### Risk/benefit of CAM use

Many parents hold the belief that in order to use CAM for their children, the modalities should be absolutely safe and beneficial. A six-year-old boy with medulloblastoma experienced adverse effects of chemotherapy. The boy received acupuncture that helped relieve chemotherapy-related vomiting. Based on this case, Gilmore [45] discussed the clinician’s responsibility to inform the parents or patients about potential beneficial CAM modalities for adverse effects of conventional cancer treatments. According to Tautz et al. [46], many parents fear the consequences of conventional cancer treatment for their child and raise the need for information on supportive CAM modalities. The parents in this study wanted to know whether CAM was safe, had less adverse effects, and was equally effective compared to conventional medicine. According to the Academy of American oncologists, parents should be advised about the indirect harm that may be caused by the financial burden of CAM and other unanticipated costs, such as the time investment required to administer the modality [43].

## Discussion

### Summary of evidence

This review demonstrates that parents of children with cancer want high quality and reliable information on CAM from authoritative sources provided at the hospital where their children are treated. Parents want this information primarily from conventional health care personnel. They emphasize autonomy for the family when making CAM treatment decision for their children. To have some sense of control of their children’s recovery and do everything possible for them, they demanded unbiased information from scientific sources to act freely on that information. In addition, they wish an open, non-judgmental conversation about CAM with the attending physician or oncologist who respect their choice. To provide ethical care and prevent decline of conventional cancer treatment, conventional health care providers need to update their knowledge about CAM, discuss risks and benefits of CAM with parents and be aware of the philosophical underpinnings of the parents’ viewpoints.

### Limitations

This integrative review must be read in light of its limitation, which largely concerns the search methodology such as the keywords and MESH term used, language limitations and scope. Generally, the variation in key terms and concepts regarding parents’ information and communication needs on CAM may have possibly missed some relevant papers pertinent to the study. Likewise, limiting studies to English, German, Dutch, and the Scandinavian languages could have missed useful papers in other languages. However, the combination of a clearly articulated (PCC) mnemonic and search methods, including a research librarian in the research team and reviewing articles with multiple experts, as well as applying critical appraisal tools to measure the methodological quality, contributed to counteract the obvious limitations of performing a systematic review. Another limitation of this review is the inclusion of studies with varying designs and study quality, which may have affected the study outcome. However, the integrative design of this review allowed for inclusion of studies of different designs, and studies of low quality were excluded from this integrative analysis.

## Conclusion

In this integrative review, the communication and information needs on CAM of parents who have children with cancer were systematically investigated. Of 24 included papers, 21 were of medium to high quality and therefore of potential use to inform paediatric oncology policy, clinical practice, and stakeholders in the field. The five main themes identified were consistent within the theories of Frankel and Stub on risk communication and information on CAM use in cancer care [52, 53], namely, information, communication, and informed decision-making.

### Information

Parents wanted to know whether CAM was safe, had less adverse effects, and was equally effective compared to conventional medicine. However, they mostly obtained CAM information from less authoritative sources such as family, friends, and the media. They expressed a need for good quality information from evidence-based sources provided at the hospital where their children received treatment (oncologists, physicians, or nurses). Many CAM modalities have not been investigated in a rigorous scientific manner and information available in the medical literature on CAM use may be inaccessible and difficult to interpret for the lay public [31]. Moreover, adverse effects of CAM and interaction with conventional care have been reported [54–56]. Advance status of the child’s disease, high risk of death at diagnosis, and influence of family and friends are significant factors in choosing CAM and may also mitigate against a truly free choice [31]. Despite these concerns, few parents in this review perceived the quality of life of their child to be compromised by using CAM, in fact most of them felt that it had improved. The American Academy of Paediatrics [43] recommends clinicians to guide parents and help them seek and assess the merits of specific CAM modalities.

### Communication

Parents emphasize an open communication about CAM with health care providers as this may avert feelings of frustration and powerlessness that impel families to these modalities. By respecting the parents’ choice and their needs to try everything for their children, health care providers provide ethical care. However, healthcare providers may need to delve more deeply into the philosophical underpinnings of the parents’ viewpoints, as fear of negative reactions from the treating oncologist/physician was found to be a major reason for parents to not disclose their children’s CAM use. According to the American Academy of Paediatrics [43], clinicians should avoid dismissal of CAM in ways that communicate a lack of sensitivity or concern for the family’s perspective. One should avoid angry and defensive reactions and seek ethical consultation in difficult cases. The refusal of conventional cancer treatment in favour of CAM is rare in Western countries, as these modalities are often used complementary [57]. Paediatricians from developing countries often face the challenge of convincing families who believe in the use of CAM alone [8].

### Informed decision-making

Results from this review demonstrate that the autonomy of the family is important when making treatment decisions for children, as adequate understandable information may empower parents and give them freedom to act on that information [31]. It is therefore of vital importance that health care providers build trustworthy and resilient relationships with parents to support them in decision-making [46], something parents of children with cancer demands, according to data from this review. Providing health–related information to patients and their relatives can empower them to make informed decisions concerning prevention, screening, and treatment [58]. It is also regarded as an essential element of high quality care [59]. A systematic review from 2015 [59], that investigated the information needs independent of certain diseases in patients and relatives in Germany, found that information about cancer treatment and juvenile rheumatic arthritis were the most prominent topics of interest. Information on CAM and nutrition was also of high interest. In a study on the demand for CAM among cancer patients, almost half of the participants who were interviewed demanded consultations about CAM, irrespective of whether they already used CAM [60]. Only a minority was not interested in CAM.

### Implication for clinical practice and further research

Based on the results of this integrative review, parents often receive advice from family and friends to choose CAM as supportive care for their children with cancer, to treat adverse effects of conventional cancer treatment or to promote quality of life and to possibly “cure” the cancer process itself. Parents express a need for high quality information on CAM from authoritative sources, preferably from healthcare professionals. These findings stress the importance to develop evidence-based decision tools on CAM use, to enable health care professionals and parents of children with cancer to make well informed, individual decisions concerning CAM.

## Data Availability

NA

## Abbreviations

CAM: Complementary and alternative medicine
